# The Role of Allografts in Revision ACL Reconstruction

**DOI:** 10.3390/medicina61081350

**Published:** 2025-07-25

**Authors:** Antonio Maestro, Carmen Toyos, Nicolás Rodríguez, Iván Pipa, Lucía Lanuza, Filipe Machado, César Castaño, Santiago Maestro

**Affiliations:** 1 Hospital Begoña, 33204 Gijón, Spain; 2 Hospital de Jarrio, 33795 Jarrio, Spain; carmentoyos@hotmail.com; 3 Hospital Cruz Roja de Gijón, 33202 Gijón, Spain; rodriguezgcia@gmail.com; 4 Hospital Universitario de Cabueñes, 33394 Gijón, Spain; pipa85@msn.com (I.P.); lanuza.lucia@gmail.com (L.L.); 5 Unidade Local de Saúde da Arrábida, 2910-446 Setúbal, Portugal; efcmachado@gmail.com; 6 Clínica JLM, 33205 Gijón, Spain; cesarcastanofernandez@gmail.com; 7 Hospital Virgen de la Concha, 49022 Zamora, Spain; smaestro17@gmail.com

**Keywords:** anterior cruciate ligament, anterolateral ligament, arthroscopy, knee, sports medicine, allograft, revision surgery

## Abstract

*Background and Objectives*: Although the use of allografts in revision anterior cruciate ligament reconstruction is associated with theoretical advantages, it has historically led to poorer clinical results and lower survival rates. However, the heterogeneity of the available literature makes it difficult to elucidate the effectiveness of allographs, as most of the studies published do not make any reference to some of the key aspects related to the processing of the allograft employed. The present study analyzed the clinical results and the survival of allografts in patients undergoing revision anterior cruciate ligament reconstruction with a well-characterized, single type of allograft. *Materials and Methods*: This was a retrospective observational study analyzing a series of patients undergoing revision anterior cruciate ligament reconstruction with an Achilles tendon allograft with a bone block (FlexiGraft, LifeNet Health), subjected to low-dose irradiation at dry ice temperatures. Preoperative and follow-up clinical variables (IKDC, pain, hop test, and YBT scores) were recorded. Survival was analyzed using the Kaplan–Meier methodology. *Results*: A total of 39 patients (34 male, 5 female) were included in the study. The mean patient age was 37.3 years and mean postoperative follow-up was 78.7 months. Forty-one percent of patients were competitive athletes, and all of the patients in the sample exhibited preoperative instability. The mean allograft thickness was 9.2 mm. During surgery, 51.3% of patients required meniscus repair and 20.5% had to be treated for chondral defects. At the last follow-up visit, 92.3% of the subjects presented with IKDC grade A and 7.7% with IKDC grade B. The mean subjective IKDC score was 0.79 and mean pain intensity was 1.15 according to the VAS scale. Limb symmetry, as measured by the various hop tests and the Y balance test, were within the safety range, with 74.4% of patients succeeding in returning to their previous level of sport. Ten-year survival was estimated at 97.4%. *Conclusions*: Allografts obtained and processed following the current regulations governing patient selection and graft harvesting, which are additionally processed without recourse to chemical procedures and sterilized at less than 2 MRad in dry ice conditions, represent an effective and safe alternative in revision anterior cruciate ligament reconstruction.

## 1. Introduction

Despite being one of the most commonly performed orthopedic procedures in the world, there are certain aspects about anterior cruciate ligament (ACL) reconstruction that still generate some controversy [1], with revision ACL reconstructions giving rise to even greater controversy [2]. One of the most frequently debated issues concerns the selection of the most appropriate type of graft, as well as the role that allografts may play in such reconstructions [3,4,5,6].

The advantages and drawbacks of allografts are well known. On the positive side, they are not associated with the risk of donor site morbidity [5,7,8], they reduce operating-room time and postoperative pain [5,8,9], they decrease the risk of iatrogenic damage, and they result in a better cosmetic result [8,10]. Moreover, allografts can be thicker than autografts, which makes them particularly useful in revision or multiple-ligament reconstructions [11]. On the other hand, allografts are associated with higher costs [5,7,12], the potential of disease transmission [5,8,12], the risk of triggering a host immune response [8], delays in biologic incorporation [7], and a higher failure rate as compared with autografts [8].

It is certainly no easy task for a surgeon to weigh the pros and cons of allograft use. This is particularly true given the heterogeneity that characterizes the studies available, notably in terms of the mechanical strength—and therefore, the failure rate—associated with allografts.

Several parameters have been identified as critical for the mechanical strength and clinical outcomes associated with the use of autografts and allografts [3]. However, given that knowledge about these parameters has developed over time, many previous studies have failed to control these parameters or to report them in sufficient detail, which limits the generalizability and clinical applicability of their findings [13]. Some of these parameters include the processing of tissues before irradiation, the target dose, and the ideal irradiation temperature [14,15,16]. Other variables that ought to be properly managed include donor demographics, the graft’s harvest site, and the postoperative rehabilitation program prescribed [5,7,17]. These oversights and lacunae in the literature are reflected in clinical practice, as it has been demonstrated that even surgeons who routinely use allografts are unaware of the techniques that result in the most effective irradiation results [16].

The question therefore remains whether the higher failure rates reported in the literature are attributable to the allograft itself or to the combined effect of a series of poorly controlled factors [16]. Various authors have suggested that low-dose and low-temperature gamma irradiation can achieve effective terminal sterilization without compromising an allograft’s mechanical strength [14,16,18,19,20,21]. In fact, in a metanalysis of 32 studies on revision ACL reconstructions, Grassi concluded that, if irradiated grafts were excluded, the outcomes of autografts and allografts were comparable [4].

The motivation for the present study stems from the need to provide more robust and clinically relevant evidence on the use of Achilles tendon allografts in revision ACL reconstruction, using a standardized graft type and processing protocol.

The purpose of the present study was to analyze the clinical and survival outcomes of a series of patients undergoing revision ACL reconstruction with a single type of allograft, providing a detailed description of its characteristics to allow an accurate evaluation of its results. By addressing some of the methodological limitations of previous investigations, we aim to contribute the data required to inform surgical decision-making in this complex clinical scenario.

## 2. Materials and Methods

This was a retrospective observational study approved by the Drug Research Ethics Committee of the Principality of Asturias (Spain) on 16 March 2020 (approval code: 2020.111). All the subjects gave their informed consent before inclusion. The study population was made up of all the patients between 18 and 55 years of age undergoing revision ACL reconstruction surgery at the Begoña hospital between 1 January 2000 and 31 December 2021, all of whom received the same type of allograft and agreed to participate.

Patients were first offered an Achilles tendon allograft. This option was preferred to reduce donor-site morbidity in revision cases where no suitable ipsilateral graft was available—since the authors favor hamstrings over other options—or to avoid additional muscle weakness in athletes whose operated limb was already compromised. Although other graft options such as hamstring or BTB allografts are also valid, the Achilles tendon allograft was chosen because its bone block allows secure tibial fixation without the need for implants, and its soft tissue portion provides sufficient thickness and length to facilitate anterolateral ligament augmentation when required. If not suitable or declined, a contralateral autograft was proposed as a second option, followed by a homolateral autograft as a last resort. Patients underwent either one- or two-stage revision depending on the diameter and condition of the previous tunnels [22]. Anterolateral reinforcement was performed in cases presenting severe rotational instability [23]. Meniscal and chondral injuries were treated intraoperatively when necessary. Standard antibiotic prophylaxis with intravenous cefazolin was administered 30 min prior to incision and continued for 48 h postoperatively. All patients underwent preoperative magnetic resonance imaging (MRI) to assess graft integrity, tunnel positioning, and potential associated intra-articular lesions. In cases where significant tunnel enlargement was suspected, computed tomography (CT) was additionally performed to obtain a more precise evaluation of tunnel size and morphology.

Apart from demographic variables (age, sex, height, weight, etc.), information was collected regarding the mechanism of injury, comorbidities, previous surgeries, Lachman [24] and pivot shift [25] scores, Tegner [26] activity level, range of motion, type of graft used, tunnel and graft thickness, additional surgical maneuvers performed, complications, pain as measured by the VAS scale, reconstruction failures, hop test scores (single, triple, crossover and 6 m timed hop test) [27], Y balance test (YBT) score [28,29,30,31], and subjective and objective IKDC scores [32]. All of this data was gathered preoperatively, except for the functional assessment scores, which were obtained at the last follow-up visit.

### 2.1. The Allograft

**Figure 1:**
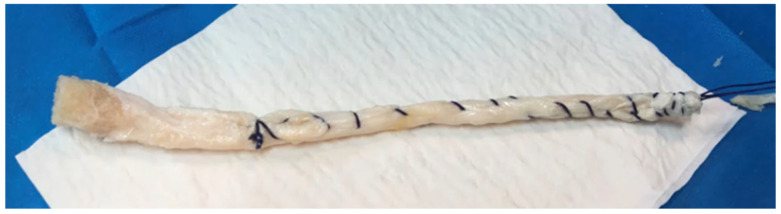
Intraoperative image of the graft, already prepared from an Achilles tendon allograft with a bone block (FlexiGraft, LifeNet Health, Virginia Beach, VA, USA).

All the allografts analyzed in this study were Achilles tendon allografts with a bone block (FlexiGraft, LifeNet Health, Virginia Beach, VA, USA), processed using the patented Allowash XG technology (LifeNet Health, Virginia Beach, VA, USA) (Figure 1). As per the manufacturer’s instructions, all donors must be subjected to a selection process characterized by more rigorous requirements than those imposed by the Food and Drug Administration (FDA) and the American Association of Tissue Banks (AATB), with donations being performed in aseptic conditions. Following a comprehensive microbiological analysis, blood elements—including bone marrow and lipids—are solubilized and removed from the tissue by means of hypotonic solutions and antimicrobial reagents combined with ultrasonication and centrifugation processes. The procedure is completed by applying a controlled dose of gamma irradiation (lower than 2 MRad) administered at dry ice temperature. The final product must be stored at a temperature ranging between −40 °C and −80 °C.

### 2.2. Graft Preparation

The Achilles tendon graft is prepared for combined anterior cruciate ligament and anterolateral ligament reconstruction on a separate surgical table. The bone block is shaped into a conical plug to facilitate tibial fixation without the need for implants. The excess soft tissue portion is trimmed and fashioned into a cylindrical graft approximately 10 mm in diameter, with the distal end slightly tapered to ease passage during reconstruction. Sutures are attached to aid in graft handling and positioning.

### 2.3. Statistical Analysis

A descriptive analysis of the data was carried out using central tendency and dispersion measures. Survival was analyzed using the Kaplan–Meier methodology and taking failure for any reason as an endpoint. Statistical significance was set at a *p* value of 0.05 in all cases. The analysis was conducted using the R statistical package (R Development Core Team, Vienna, Austria), version 4.5.0 [33].

## 3. Results

The study comprised a total of 38 patients (33 male and 5 female), with a mean age of 36.4 ± 9.7 years and a mean body mass index of 24.8 ± 2.7 kg/m^2^. Sixteen (42.1%) subjects presented with a Tegner score of 7 or more; i.e., they played sports at a competitive level, with three of them being professional athletes. All primary ACL reconstructions in this series were performed using a single-bundle technique with hamstring autografts. Regarding the causes of failure leading to revision surgery, 24 cases were related to sports injuries, 10 to accidents, 1 was atraumatic, and 1 occurred during activities of daily living not associated with sports or trauma. Before surgery, all patients exhibited some degree of anteroposterior instability, as measured by the Lachman scale, and of rotational instability, as evaluated by the pivot-shift test. The patients’ anthropometric and baseline data are shown in Table 1.

The mean allograft thickness was 9.22 mm (range: 8–10 mm), and 50.0% (N = 19) of patients were subjected to meniscus repair during the procedure. A total of 21.1% (N = 8) of subjects were treated for chondral defects. Intraoperative complications arose over the course of four of the procedures (10.5%).

**Figure 2:**
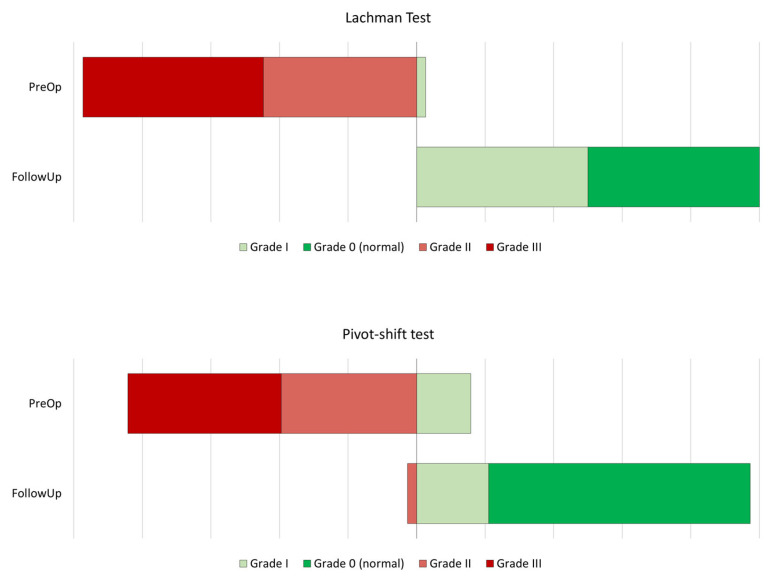
Progression of the Lachman and the pivot-shift scores from preoperative to the last follow-up visit.

The mean follow-up was 79.2 months (range: 42–124 months). At the last follow-up visit, 35 (92.1%) patients presented with a normal IKDC score (grade A), while in three (7.9%) cases the IKDC score was found to be nearly normal (grade B). The mean subjective IKDC score was 0.80 ± 0.07, and the mean pain intensity, as measured by the visual analog scale, stood at 1.18 ± 1.8. Limb symmetry was 95.8 ± 13.0% on the single hop test, 95.3 ± 10.9% on the triple hop test, 96.7 ± 25.4 on the crossover hop test, 104.0 ± 12.9% on the 6 m timed hop test, and 98.7 ± 7.9% on the composite YBT. As regards the patients’ return to sports, 28 (73.7%) succeeded in going back to their preoperative performance level. Additionally, we examined whether the presence of meniscal or chondral damage, as well as residual pivot shift, influenced subjective outcomes or return to sport. In this regard, the presence of a meniscus (*p* = 0.393) or a chondral lesion (*p* = 0.090) did not appear to significantly influence patient IKDC scores. However, return-to-sport rates were significantly lower in patients with chondral damage (*p* = 0.019), meniscal damage seemingly not exerting a significant effect (*p* = 0.269). The various measurements recorded at the last follow-up visit are represented in Table 2, while the patients’ performance on the different tests conducted over time is depicted in Figure 2.

**Figure 3:**
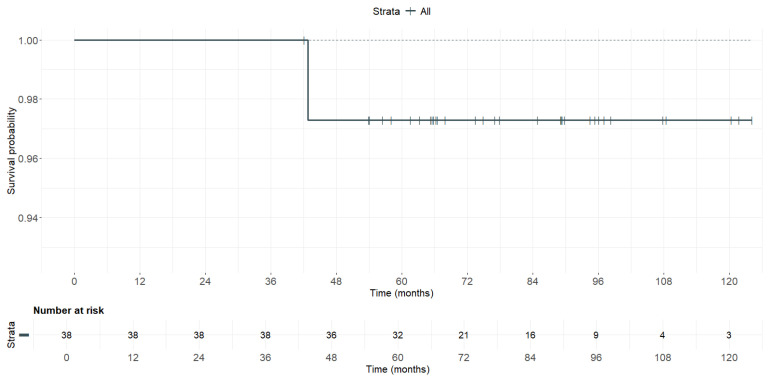
Kaplan–Meier graph illustrating the survival of the allografts used in the series.

The only re-rupture in this series occurred due to a subsequent traumatic event. Survival, as calculated by the Kaplan–Meier method (Figure 3), was estimated at 97.3% (95% CI: 92.2–100%) at 10 years from surgery.

## 4. Discussion

Although many advantages have been associated with the use of allografts in revision ACL reconstruction [5,7,8,10,11], three of them are in our opinion of particular significance. The first one is their ability to offer patients an alternative in the foreseeable absence of a homolateral autologous graft. The second one lies in the fact that the Achilles tendon is sufficiently thick [8,10,11] and sufficiently long to also allow reconstruction of the anterolateral ligament during the same procedure, which has been shown to improve rotational stability [22,23,34,35,36,37]. The third advantage has to do with the fact that state-of-the-art processing methods can achieve a sterility assurance level (SAL) of 10^−6^ without undermining the allograft’s mechanical strength [14,16,18,19,20,21]. In fact, the disease transmission rate in these surgeries currently stands below 0.015% [16,38], with autografts and allographs exhibiting comparable infection rates [39]. Of course, this does not entail the superiority of allografts or suggest that allografts are the only option available for patients undergoing revision ACL reconstruction. Autologous grafts—such as those from the contralateral leg or the quadriceps tendon, as well as those of the bone-patellar tendon-bone type—are equally valid options, particularly in the revision setting. Our intention is simply to present allografts as one additional tool in the surgeon’s armamentarium.

Despite the above, however, the use of allografts has historically been considered a suboptimal solution associated with severe limitations. Although some of these limitations have been objectively ascertained, others are related to problems arising from the various conservation or sterilization procedures applied to allografts, which have been unsystematically reported in the literature, generating no small amount of uncertainty [5]. In this regard, even if several studies appear to demonstrate that allografts are associated with poorer survival rates and/or poorer clinical results than autografts [40,41,42,43,44,45,46,47,48], an increasing number of authors have pointed out that this could be due to such factors as the use of suboptimal allograft processing or sterilization methods which, if taken in due consideration, would render the differences between autografts and allografts statistically nonsignificant [4,8,17,49,50,51]. In fact, several analyses have suggested that gamma irradiation interferes with allografts’ biomechanical properties in a dose- [52] and temperature-dependent [53,54] manner. Similarly, physical or chemical pre-sterilization processing also affects an allograft’s properties [7,8]. These findings, however, have for the most part been ignored in studies comparing the results of allografts with those of autografts [5]. In this respect, Sikka et al. [13] reported that only 38% of the studies on the subject made mention to tissue banks and only 69% described the processing method used. This lack of standardization and detail makes it impossible to determine whether the unfavorable clinical results observed are due to the allograft itself or to the processing method employed [5,16]. It should therefore come as no surprise that a survey by the American Orthopedic Society for Sports Medicine (AOSSM) revealed that 20% of surgeons did not know whether the allografts they used had been irradiated or not, and two-thirds were not aware of the dose required to ensure proper sterility [16].

Current reports in the literature claim that low-dose gamma irradiation, performed in a controlled way and at low temperatures, results in effective terminal sterilization without undermining the allograft’s mechanical strength [14,16,18,19,20,21]. The safe threshold irradiation dose has been set at 2 MRad, as studies have shown that the effect of gamma rays on the tissue’s biomechanical properties is negligible below this value [14]. As regards temperature, it has been demonstrated that allografts irradiated at dry ice temperatures (–78.5 °C) are less brittle and that their collagen is less prone to damage, with less generation and diffusion of free radicals [53,54]. In this regard, Balsly et al. [18] did not find changes in the mechanical strength or the elasticity modulus of allografts treated with low-dose gamma irradiation (1.83–2.18 MRad) at dry ice temperatures. Similarly, Greaves et al. [19] demonstrated that low-dose (1.46–1.80 MRad) and low-temperature irradiation does not affect the allograft’s maximum strength. Aguila et al. [20] found no statistically significant differences in elongation, ultimate load, or ultimate stress between allografts irradiated at low doses (1.5–2.5 MRad) and those not subjected to irradiation. This threshold dose is also applicable in clinical practice, as demonstrated by a study on 5968 patients where failure rates were significantly higher when allografts were sterilized at doses above 1.8 MRad [21]. Nevertheless, it remains to be clarified whether preservation of the allograft’s biomechanical properties plays any role in the clinical success of ACL reconstruction [52].

Another factor that could bias the analysis has to do with the fact that studies comparing irradiated and non-irradiated allografts tend to include a sundry collection of various allograft preparations in the latter category, which could lead to an inaccurate evaluation of processing techniques and of clinical outcomes [16]. Indeed, allografts may be prepared using mechanical lavage, freezing, radiation, chlorhexidine, or carbon dioxide, and each of these techniques affects their structural and mechanical properties in a different way [7,8]. In fact, some tissue providers have been found to pre-process “non-irradiated” or “non-sterilized” allografts by subjecting them to irradiation doses of 1.0–1.5 MRad for safety reasons [14].

A comparison of the results of the present series with those of a recent metanalysis that only included autograft revisions [55] did not reveal any significant differences. Specifically, our patients achieved a subjective IKDC score of 80.0 points, which represents a minimal clinical difference with respect to the 81.4 points reported in the said metanalysis. Pain scores were also comparable (1.18 in our series as compared with 1.26 in the metanalysis), and the same can be said about the results of the pivot-shift test (81.8% negative, 14.1% slightly positive, 3.1% moderately positive, and 1.0% severely positive in the metanalysis, as compared with 76.3%, 21.1%, 2.6%, and 0.0%, respectively, in our study. The greatest difference was related to the mean failure rate, which was as high as 11.83% in the metanalysis and a mere 2.6% in this study.

It is also interesting to compare our data with that of a previous metanalysis [56] of revision ACL reconstructions. Although that metanalysis included allografts in only 7% of cases, the surgical technique employed included an anterolateral reinforcement, as did most of the cases in our series (N = 33; 84.6%). The authors in the metanalysis obtained a slightly higher subjective IKDC score (83.3 points) and very similar pivot-shift scores to those in this analysis (83% grade 0, 15% grade I, 1% grade II, and 1% grade III). Return to sports at the same or higher level in the metanalysis was 41%, significantly below the 73.7% rate observed in our series.

A recent systematic review highlighted the importance of graft selection and processing techniques for the outcome of ACL reconstruction [57]. The difference in autograft and allograft failure rates appears to be influenced not only by graft type but also by the preservation and sterilization methods employed. For instance, high-dose gamma irradiation (>1.8 MRad) has been identified as a significant predictor of revision surgery, whereas low-dose irradiation (1.2 MRad) does not seem to compromise graft integrity. Similarly, of the various cleansing techniques available, only BioCleanse has been associated with a higher risk of failure, while AlloWash and AlloTrue have not been shown to exert any influence on the ACL tear rate. These findings warrant detailed reporting and standardization of allograft processing protocols in clinical studies, as the variability in preparation methods may confound comparisons between graft types. Also, a study by Kentel et al. [58]. found comparable outcomes between semitendinosus autografts and allografts in single-stage ACL revision surgery, with no significant differences in knee stability, return to sport, or patient-reported outcomes. These findings suggest that, when properly selected and processed, allografts may be a valid alternative to autografts in the revision setting.

Regarding the re-rupture rate, our series demonstrates a 10-year survival rate of 97.3%, as estimated using the Kaplan–Meier methodology. This figure is consistent with those reported in a recent systematic review on revision procedures performed using autografts [59], in which the proportion of re-ruptures (range, 0–25%) exceeded 5% in only four out of sixteen series, and surpassed 10% in just one of them.

The data obtained from our analysis is in line with that from recent studies that have found that, if properly selected, harvested, and processed, allografts may result in success rates comparable to those of autografts [4]. In this respect, it is now commonly accepted that the results of ACL reconstructions using a properly processed allograft in over-25-year-old or less athletic patients are as good as those obtained when autografts are used [60,61].

The present study presents limitations inherent in its retrospective design and its small sample size, which warrant careful interpretation of the results. The absence of a control group and the fact that the study was conducted at a single center with a high proportion of competitive athletes may limit the generalizability of the findings. Also, the statistical analysis was primarily descriptive, as subgroup or multivariate analyses were unfeasible due to the limited sample size. However, this study constitutes one of the very few clinical studies published on revision ACL reconstructions, and it provides a thorough description of allograft processing and sterilization techniques. This should contribute to more accurate standardization of the relationship between the characteristics of the allografts used and the clinical results obtained, thereby reducing the heterogeneity currently observed in the literature.

## 5. Conclusions

Allografts obtained following the current patient-selection and graft-harvesting regulations, which are processed without recourse to chemical procedures and sterilized at doses lower than 2 MRad at dry ice temperatures, represent an effective and safe alternative for revision ACL reconstruction.

## Figures and Tables

**Table 1:** Anthropometric and baseline data of the patients in the study.

Variable	N	%	Mean	SD	Minimum	Maximum
Sex						
Males	33	86.8%	-	-	-	-
Females	5	13.2%	-	-	-	-
Laterality						
Right	21	55.3%	-	-	-	-
Left	17	44.7%	-	-	-	-
Age	38	-	36.4	9.7	18	53
Height	38	-	174.7	7.2	153	193
Weight	38	-	75.9	9.5	50	93
Body mass index	38		24.8	2.7	19.3	31.5
Tegner score—PreOp						
Lower than a 7	22	57.9%	-	-	-	-
Equal to or higher than 7	16	42.1%	-	-	-	-
Lachman test—PreOp						
Grade I	1	2.6%	-	-	-	-
Grade II	17	44.7%	-	-	-	-
Grade III	20	52.6%	-	-	-	-
Pivot-shift—PreOp						
Grade I	6	15.8%	-	-	-	-
Grade II	15	39.5%	-	-	-	-
Grade III	17	44.7%	-	-	-	-

**Table 2:** Data at the last follow-up visit.

Variable	N	%	Mean	SD	Minimum	Maximum
Follow-up time	38	-	79.3	20.5	42	124
Lachman test—FollowUp						
Normal	19	50.0%	-	-	-	-
Grade I	19	50.0%	-	-	-	-
Pivot-shift—FollowUp						
Normal	29	76.3%	-	-	-	-
Grade I	8	21.1%	-	-	-	-
Grade II	1	2.6%	-	-	-	-
IKDC Score—FollowUp						
A—Normal	35	92.1%	-	-	-	-
B—Nearly normal	3	7.9%	-	-	-	-
Subjective IKDC—FollowUp	38	-	0.80	0.07	0.57	0.95
Pain (VAS)—FollowUp	38	-	1.18	1.8	0	7
Single hop test—LSI	38	-	95.8%	13.0%	58.9%	135.0%
Triple hop test—LSI	38	-	95.3%	10.9%	58.7%	111.4%
Crossover hop test—LSI	38	-	96.7%	25.4%	81.3%	203.7%
6 m timed hop test—LSI	38	-	104.0%	12.9%	77.8%	148.0%
Composite YBT score—LSI	38	-	98.7%	7.9%	81.8%	131.5%
Return to sport						
Same level or higher	28	73.7%	-	-	-	-
Could not reach same level	10	26.3%	-	-	-	-

## Data Availability

Datasets are available on request from the authors.

## Abbreviations

The following abbreviations are used in this manuscript:

IKDC	International Knee Documentation Committee
YBT	Y Balance Test
ACL	Anterior Cruciate Ligament
FDA	Food and Drug Administration
AATB	American Association of Tissue Banks
VAS	Visual Analog Scale for Pain
